# Supplementary material for: First occurrence of the enigmatic peccaries Mylohyus elmorei and Prosthennops serus from the Appalachians: latest Hemphillian to Early Blancan of Gray Fossil Site, Tennessee
Source: PeerJ. 2018 Nov 30;6:e5926. doi: 10.7717/peerj.5926 (PMC6276594; doi:10.7717/peerj.5926)
Supplement: Table S1 — All specimens are listed based on their locality and the publication from which they were initially described. [file peerj-06-5926-s001.docx]

Supplementary Table 1. Specimens of *Mylohyus elmorei* and *Prosthennops serus* referenced in the text.

*Mylohyus elmorei*

Palmetto Fauna (White, 1942; Wright & Webb, 1984)

UF/TRO 412 (TRO F7701N): partial ramus with R. p3-m3

UF 12265: partial cranium with bearing R. P3-M3 and L. M2-M3

UF 57280 (MCZ 3805): cast of partial ramus with L. p2-m3

UF 294749: isolated R. p4

*Prosthennops serus*

Tyner Farm Locality (Hulbert et al., 2009a)

UF 212306: partial mandible with i1, c1, and p2-m3

Ft-40 Locality (Schultz & Martin, 1975)

UNSM 76052: partial mandible with L. i2-c1, p2-m3 and R. i3, c1, p2-m3

UNSM 76059: partial mandible with L. i1-i2, c1, p2, and p4-m3 and R. i1-i2 and c1

Gd-10 Locality (Schultz & Martin, 1975)

UNSM 76054: partial mandible with L. i1-c1 and R. i1-i2, canine, and p2-m3
